# Network analysis of risk factors for firearm assault

**DOI:** 10.1007/s10865-026-00698-8

**Published:** 2026-08-31

**Authors:** Dorothy R. Stearns, Patrick M. Carter, Philip Stallworth, M. Kit Delgado, Lauren K. Whiteside, Erin E. Bonar, Jason E. Goldstick

**Affiliations:** 1 Institute for Firearm Injury Prevention, University of Michigan, 1109 Geddes Ave, Ann Arbor, MI USA; 2 Injury Prevention Center, University of Michigan, 2800 Plymouth Road, Suite B10-G080, Ann Arbor, MI USA; 3 Department of Emergency Medicine, University of Michigan, 1500 E Medical Center Drive, Ann Arbor, MI USA; 4 Department of Health Behavior and Health Education, University of Michigan, 1415 Washington Heights, Ann Arbor, MI USA; 5 Department of Emergency Medicine, University of Pennsylvania, Philadelphia, PA USA; 6 Department of Emergency Medicine, University of Washington, 325 9th Ave, Box 359702, Seattle, WA 98104 USA; 7 Michigan Innovations in Addiction Care Through Research and Education Program, Department of Psychiatry, Addiction Center, University of Michigan, 2800 Plymouth Road, Building 16 Floor 2, Ann Arbor, MI USA

**Keywords:** Network analysis, Firearm assault, Risk factors, Young adults, Community violence

## Abstract

Although individual risk and protective factors have been identified, the extent to which these factors interact within a broader risk network remains inadequately understood. We used network analysis to characterize the partial correlation structure among previously identified risk factors for firearm violence. We used baseline data from a prospective longitudinal study of 1506 participants aged 18–24 recruited from four Level 1 trauma centers in Philadelphia, Flint, and Seattle (2021–2023), 14.4% of which self-reported firearm assault (victimization or aggression, including threats) at baseline. Risk factors included substance use, mental health symptoms, firearm carrying, peer/family factors, and community factors. Regularized partial correlation network models estimated the partial correlation network. We characterized the network using various centrality indices, and discriminatory power of variable domains was quantified by the area under the receiver operator characteristic curve (AUROC). The network showed groupings of mental health symptoms (depression, anxiety, PTSD), substance use and negative behaviors (alcohol use, drug use, negative peer behaviors), and protective factors (resiliency, prosocial attitudes, peer support, positive peer behaviors, parental support). Centrality measures identified PTSD, community violence exposure, negative peer behaviors, and family conflict as strongly interconnected and influential. Nearly all predictive power of the full predictor set (AUROC = 0.86) was captured using these central factors; community violence was the strongest standalone factor (AUROC = 0.81). Firearm violence risk among young adults is shaped by an interconnected network of psychosocial, familial, and community factors. Central risk elements, particularly community violence and PTSD, emerge as high-impact leverage points for trauma-informed, multifaceted interventions addressing social and structural determinants.

## Introduction

Firearm assault injuries among young adults in the United States represent a critical public health challenge, with individuals aged 18–24 being the highest risk age group (23.9 deaths per 100,000 population) (Centers for Disease Control and Prevention, 2021). Emergency departments (EDs) and trauma centers serve as key points of contact for young people at elevated risk for subsequent violence involvement, including firearm assault victimization, threats, and aggression. Recent work showed that more than 1 in 7 young adults entering urban EDs reported recent exposure to firearm violence (as victim or aggressor, including threats) (Goldstick et al., 2024a, 2024b). These encounters create time-sensitive opportunities to identify modifiable correlates of firearm assault involvement and to guide prevention strategies targeting interconnected risk processes.

A substantial body of literature has identified key correlates of firearm violence among young adults. At the individual level, retaliatory attitudes (Goldstick et al., 2019), substance use, and mental health symptoms, including PTSD, depression, and anxiety, are consistently associated with risky firearm behaviors (Carter et al., 2015, 2020). At the interpersonal level, peer processes, such as engaging in physical aggression and affiliation with delinquent peers, are strong predictors of firearm access and possession (Mattson et al., 2020; Walsh et al., 2023). Similarly, prior violence experience, including non-firearm violence, has shown to increase rates of firearm aggression initiation (Goldstick et al., 2019). Additionally, social embeddedness in gun-carrying peer networks heightens lethal conflict risk, as firearms are often viewed as collective protection and peers frequently act as co-offenders (Wilkinson et al., 2009). Family dynamics, specifically parental support and low levels of conflict, play a critical role in mitigating risk (Ross et al., 2023; Walsh et al., 2023). Evidence from case–control studies further suggests that family structure is a significant factor, with adolescents living in households with fewer than two parents facing nearly four times the odds of sustaining a non-fatal firearm injury (Paris et al., 2002). At the community level, violence exposure is strongly associated with firearm violence involvement (Goldstick et al., 2024a, 2024b).

While specific risk factors have been identified, there remains a gap in understanding how these factors are jointly associated with one another. Importantly, risk factors for firearm violence are not independent of one another; in other words, firearm violence can be conceptualized as an element of a grouping of other factors, resulting from a complex interplay of exposures and behaviors (Jessor, 1982). Some work has quantified how violence-related factors—both risk-enhancing and protective—are interrelated (Culyba et al., 2019), though the network of interdependencies remains not-well-characterized, and prior work is not specific to firearm violence. Understanding that interdependence may help identify high-leverage points to intervene in the risk factor network.

Given that complex nature of firearm violence, network analysis of the association structure among factors related to firearm violence offers a useful approach for characterizing their interdependence. By visually representing variables as nodes and regularized partial correlations as edges, this approach highlights the most central variables and the bridges between distinct risk groupings. Network models map the entire conditional association structure, helping to generate hypotheses about how risk and protective factors co-occur. For clinical settings, where brief screenings must address a multitude of needs, identifying these highly interconnected factors is essential for prioritizing intervention and referral strategies.

In this report, we analyze baseline data from the Screening to Predict Young Adults at Risk for Firearm Violence (SPARK) project, a prospective longitudinal study of firearm violence (Goldstick et al., 2024a, 2024b). Previous work from this project (Goldstick et al., 2024a, 2024b) showed that recent firearm assault history was associated community violence, adverse childhood experiences (ACES), mental health symptoms (PTSD, depression, anxiety), family conflict, and substance use, while protective factors such as parental support, prosocial attitudes, and peer support were negatively associated. Here, we extend this work by using regularized partial correlation network models to understand and describe the association structure within the risk factor network for firearm violence. We then describe several quantifications of centrality in that network, in combination with the discriminatory power of individual factors associated with firearm violence. These findings aim to inform the development of targeted, multi-level prevention strategies for firearm violence among high-risk young adults.

## Methods

We used baseline data from SPARK, which is a prospective longitudinal study of adults age 18–24 years recruited from 2021 to 2023 in four Level-1 trauma centers in three cities (Philadelphia, Flint, and Seattle). Detailed study methods are described elsewhere (Goldstick et al., 2024a, 2024b), briefly, the overall purpose of SPARK was to externally validate the SaFETy (Serious fighting, Friend weapon carrying, community Environment, and firearm Threats) score (Goldstick et al., 2017, 2024a, 2024b), the only existing screening tool for firearm violence. Consenting participants agreed to be contacted at 6- and 12-months for follow-ups, and to prospective chart reviews for twelve months post-baseline. Those unable to consent (e.g., due to medical/cognitive incapacitation), those presenting for sexual assault or other conditions requiring intensive psychosocial services, and those under droplet precautions were ineligible. Among individuals approached, 25.4% declined participation prior to completing the baseline survey, yielding a final analytic sample of 1506 participants. Sample characteristics have been described previously (Goldstick et al., 2024a, 2024b).

## Measures

In this analysis, we focused on estimating the correlation structure between factors examined previously (Goldstick et al., 2024a, 2024b) in relation to firearm assault. In total, there were sixteen factors:

- *Substance use (2 measures)*: Alcohol use was measured by the Alcohol Use Disorder Identification Test—Consumption (AUDIT-C) sum (Bush et al., 1998). Drug use frequency was measured using items from the Alcohol, Smoking and Substance Involvement Screening Tests (ASSIST) (National Institute on Drug Abuse (NIDA), 2024). We summed the use frequencies across all nine substances: marijuana, cocaine, hallucinogens, street opioids, prescription opioids, prescription sedatives, prescription stimulants, methamphetamine, or inhalants, as in prior validation work (Humeniuk et al., 2006, 2008).
- *Mental health (3 measures)*: Depression symptoms were measured by the Patient Health Questionnaire-9 (PHQ-9) (Richardson et al., 2010) sum; anxiety symptoms were measured with the Generalized Anxiety Disorder-7 (GAD-7) (Spitzer et al., 2006) sum; PTSD symptoms were measured with the PTSD Checklist-17 (Weathers et al., 1993) sum.
- *Firearm carrying*: Past 6-month firearm carrying frequency (7-point scale; 0 = Never, 6 = 20 + times) was measured using the CDC Behavioral Risk Factor Surveillance System (BRFSS, 1-item) (Centers for Disease Control & Prevention, 2021).
- *Peer behaviors (3 measures)*: Items from the Flint Adolescent Study (FAS) (Zimmerman et al., 2002) measured positive peer behavior (e.g., college attendance; 4-item average), negative peer behavior (e.g., weapon carrying; 7-item average), and peer support (3-item average).
- *Familial factors (3 measures)*: Family conflict (5-item average) was measured with items from FAS (Zimmerman et al., 2002); parental support (6-item average) was measured using the Procidano and Heller’s social support scale (6-item average) (Procidano & Heller, 1983); and ACEs were measured as the number of ACEs out of eight items queried in the National Survey of Children’s Health (8-item total) (*The National Survey of Children’s Health*, n.d.).
- *Resiliency/Prosociality (2 measures)*: The Brief Resilient Coping Scale (4-item average) (Sinclair & Wallston, 2004) measured resiliency, and the Prosociality Scale (8-item average) (Caprara et al., 2005) measured prosociality.
- *Other (2 measures)*: We measured past-six-month community exposure to crime and violence using the “Things I’ve Seen and Heard” scale (14-item average) (Richters & Saltzman, 1990), and measured retaliatory attitudes with items from Hill and Noblin (7-item average) (Hill & Noblin, 1991), with higher scores indicating greater agreement with statements favoring retaliation.

Past-six-month firearm assault was measured from a modified version of the conflict tactics scale (CTS-2) (Straus et al., 1996); any response other than ‘Never’ on questions about firearm violence (including threats), as victim or aggressor (non-partner or partner), was counted as a positive response. Victimization and aggression were combined, as done in prior work (Carter et al., 2015; Goldstick et al., 2017, 2024a, 2024b, 2026). We further justify this specification in two ways. First, although firearm assault victimization was reported by > 90% of those with any firearm assault in this sample, there was substantial overlap between those who reported firearm assault victimization and those who reported firearm assault aggression (Goldstick et al., 2024a, 2024b). Second, we note that there is substantial overlap between risk and protective factors for violent aggression and violence victimization (Jennings et al., 2010).

### Data analysis

We estimated the partial correlation network between the above factors using the R package *lavaan* (Rosseel, 2012), with its structure penalized using the graphical LASSO to produce shrink/eliminate small inconsequential correlations (Friedman & Tibshirani, 2011) using the *qgraph* package (Epskamp et al., 2023). By focusing on partial correlations, the edges in the network reflect correlations between variables after controlling for all other variables in the network, isolating conditionally dependent associations in the visualization. The graphical LASSO penalty functions to shrink small/inconsequential partial correlations, which allows the visualization, and centrality metrics, to focus on the largest associations and reduce the influence of statistical noise. Fitting this model requires specification of a tuning parameter, γ, which dictates part of the form of the Extended Bayesian Information Criteria (EBIC) that is optimized when estimating the network. A value of γ = 0 corresponds to conventional BIC, and larger values correspond to higher penalty levels on the number of edges in the network. We specified γ = 0.5, which is generally regarded as a safe choice (Foygel & Drton, 2010) and has been used in other injury-related applications (e.g., Mattsson, 2019).

We displayed the network graphically, with edge thickness displaying the strength of the relationship (i.e., the edge weight), and the color (red: negative; green: positive) indicating the direction. To simplify the network diagram, we did not plot non-zero edges corresponding to (regularized) estimated partial correlations that were less than 0.1, which is generally regarded as a small correlation in the behavioral sciences (Cohen, 2013).

Using this network, we computed four centrality measures for each node: (1) Betweenness: how often a variable appears in the shortest path (quantified by the sum of the edge lengths) between two other nodes; (2) Closeness: the sum of the shortest paths (quantified as above) from the node to each other node; (3) Strength: the sum of the (absolute) edge weights leading out of a node; and, (4) Expected influence: similar to ‘Strength’ but using signed values, rather than absolute values. Note that although we suppressed small (< 0.1) partial correlations from the network graph, we did not *eliminate* those edges in the computation of the centrality measures When displaying centrality measures, all measures are scaled by the maximum for interpretation ease. Because the network was estimated among predictors only, centrality reflects relationships among risk factors rather than their associations with firearm assault.

One potential concern with interpretation of such centrality measures is their stability (i.e., large changes to those metrics in response to relatively small changes in the input data). To rule out this concern, we performed stability analysis by bootstrap resampling as described elsewhere (Epskamp et al., 2018). Specifically, we plotted the average correlation between node-level centrality measures (e.g., strength; betweenness) and those estimated by bootstrap resampling fractions of the data set, to determine what fraction of the sample can be dropped while still maintaining 0.7 correlation, with > 50% typically indicating good stability (Epskamp et al., 2018). To estimate those correlations we used n = 2000 bootstrap resamples at each fraction.

To contextualize the network analysis, we also examined the discriminatory power of the individual variables and different elements of the network. Specifically, we described the power of each individual covariate to discriminate between those with versus without firearm violence, measured using the Area Under the Receiver Operator Characteristic Curve (AUROC). We also examined how much discriminatory power, relative to the full model, is captured by the most central network elements. All analyses were conducted using R v4.4.1.

## Results

### Sample description

As reported previously (Goldstick et al., 2024a, 2024b), the sample included 1,506 young adults aged 18–24 years (mean age 21.3), of whom 61.4% were female, 35.9% male, and 2.7% non-conforming. The cohort was racially diverse (41.7% Black, 33.6% White, 9.8% multiracial, 7.2% Asian), and 33.1% were currently enrolled in school. Overall, 50.0% of participants reported past six-month violence involvement, including 42.5% reporting non-partner violence and 25.7% partner violence; 14.4% reported firearm assault (as victim or aggressor).

Table 1 shows the distribution of each of the sixteen measures included in the network analysis. As noted in the methods section, all scales were kept in their numeric form for the partial correlation network analysis, rather than reduction to cut points. While more detailed descriptions of the clinical characteristics are shown in prior work (Goldstick et al., 2024a, 2024b) we note here 60.5% of the sample reported risky use of alcohol (AUDIT-C score of 3 + for women and 4 + for men) or at misuse of at least one substance (NIDA-ASSIST sum 4 + for any of the nine drugs measured), and 50.2% screened positive for at least one mental health condition (PTSD: PCL-17 ≥ 30, depression: PHQ-9 ≥ 10, or anxiety: GAD-7 ≥ 10).

**Table 1:** Distribution of each variable analyzed in the network analysis

	Range	Mean (SD)	Median (IQR)	Number Missing (%)
*Peer behaviors*				
Peer positive behaviors	0–4	1.6 (0.9)	1.5 (1.0–2.5)	26 (1.7%)
Peer negative behaviors	0–4	0.7 (0.6)	0.6 (0.3–1.1)	26 (1.7%)
Peer support	1–5	3.2 (1.1)	3.3 (2.3–4.0)	9 (0.6%)
*Familial/parental*				
Parental support	1–5	3.3 (1.2)	3.3 (2.3–4.3)	18 (1.2%)
Family conflict	1–4	1.7 (0.7)	1.4 (1.0–2.0)	18 (1.2%)
Adverse childhood experiences	0–8	2.6 (2.1)	2.0 (1.0–4.0)	35 (2.3%)
*Community & attitudes*				
Community violence	0–3	0.6 (0.7)	0.4 (0.1–0.9)	40 (2.7%)
Retaliatory attitudes	1–4	2.4 (0.5)	2.4 (2.1–2.7)	32 (2.1%)
Resiliency	1–5	3.8 (0.8)	3.8 (3.3–4.3)	15 (1.0%)
Prosocial attitudes	1–5	3.8 (0.9)	4.0 (3.3–4.5)	35 (2.3%)
*Past-six-month firearm behaviors*				
Firearm carriage frequency	0–6	0.4 (1.3)	0.0 (0.0–0.0)	8 (0.5%)
*Substance use/mental health*				
Past-six-month alcohol use	0–12	2.2 (2.5)	1.0 (0.0–4.0)	11 (0.7%)
Past-six-month drug use	0–54	3.1 (4.0)	2.0 (0.0–6.0)	3 (0.2%)
Past-month PTSD symptoms	0–68	20.5	16.0 (6.0-	55 (3.7%)
Past-two-week depression	0–27	(17.2)	33.0)	27 (1.8%)
symptoms	0–21	7.7 (6.3)	7.0 (2.0–12.0)	31 (2.1%)
Past-two-week anxiety symptoms		7.9 (6.7)	7.0 (2.0–12.0)	

The ‘Range’ column refers to the hypothetical range of the entire scale, not the observed minimum and maximum in these data

### Network characteristics

**Fig. 1:**
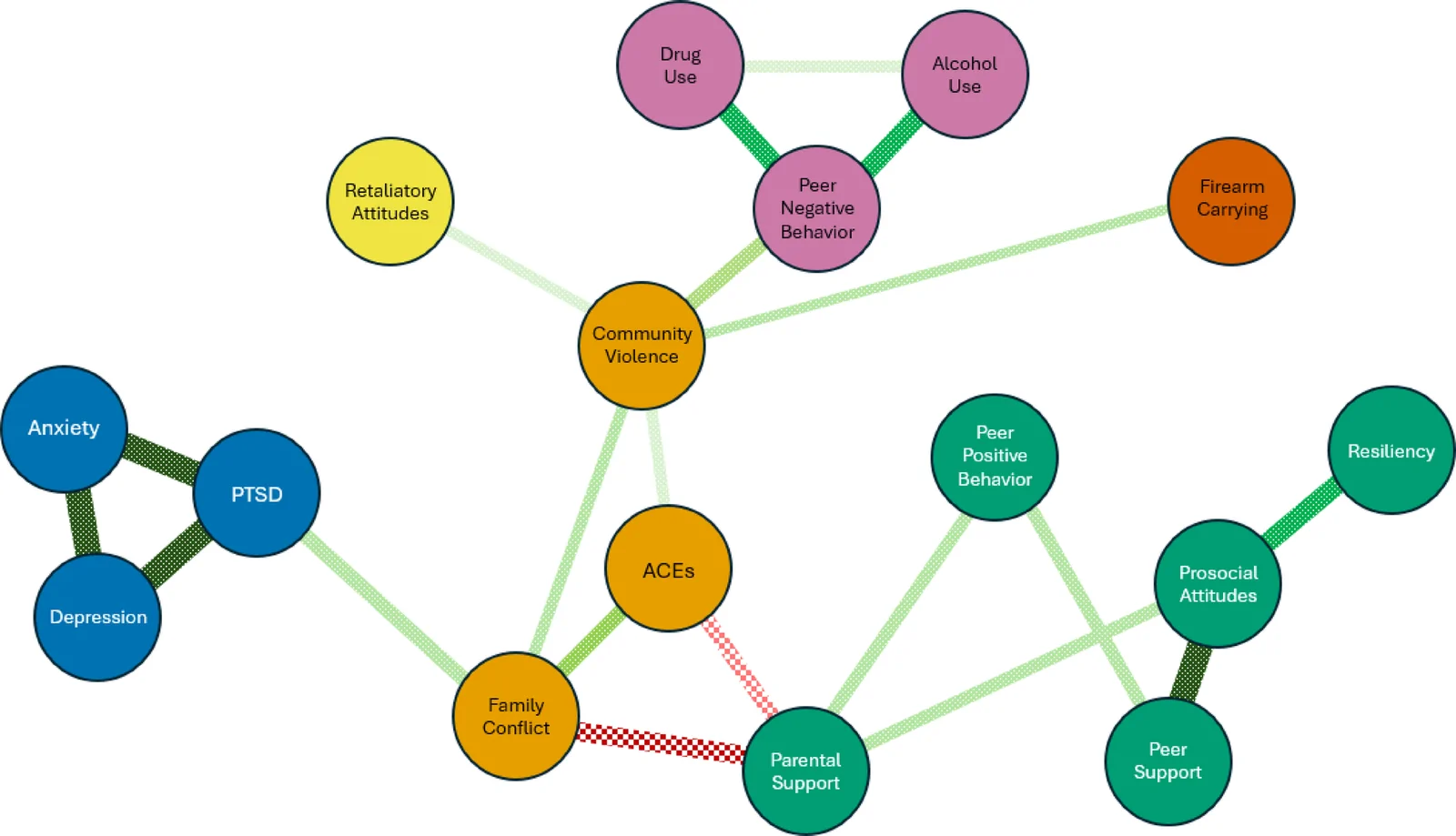
Partial correlation network between factors potentially associated with firearm violence, penalized using the graphical lasso. The partial correlation network was estimated by the graphical lasso with *γ =0.5* Red edges indicate a negative correlation, and green edges indicate a positive correlation

Figure 1 shows the regularized partial correlation network estimate. Grouped together are a mental health symptom triad (anxiety, depression, PTSD), a substance use and negative behavior triad (drug use, alcohol use, negative peer behaviors), and a protective factor grouping (resiliency, prosocial attitudes, peer support, positive peer behaviors, parental support). Firearm carrying was linked to the rest of the network through community. Community violence, family conflict, and ACEs, at least weakly, connected with multiple network sections.

**Fig. 2:**
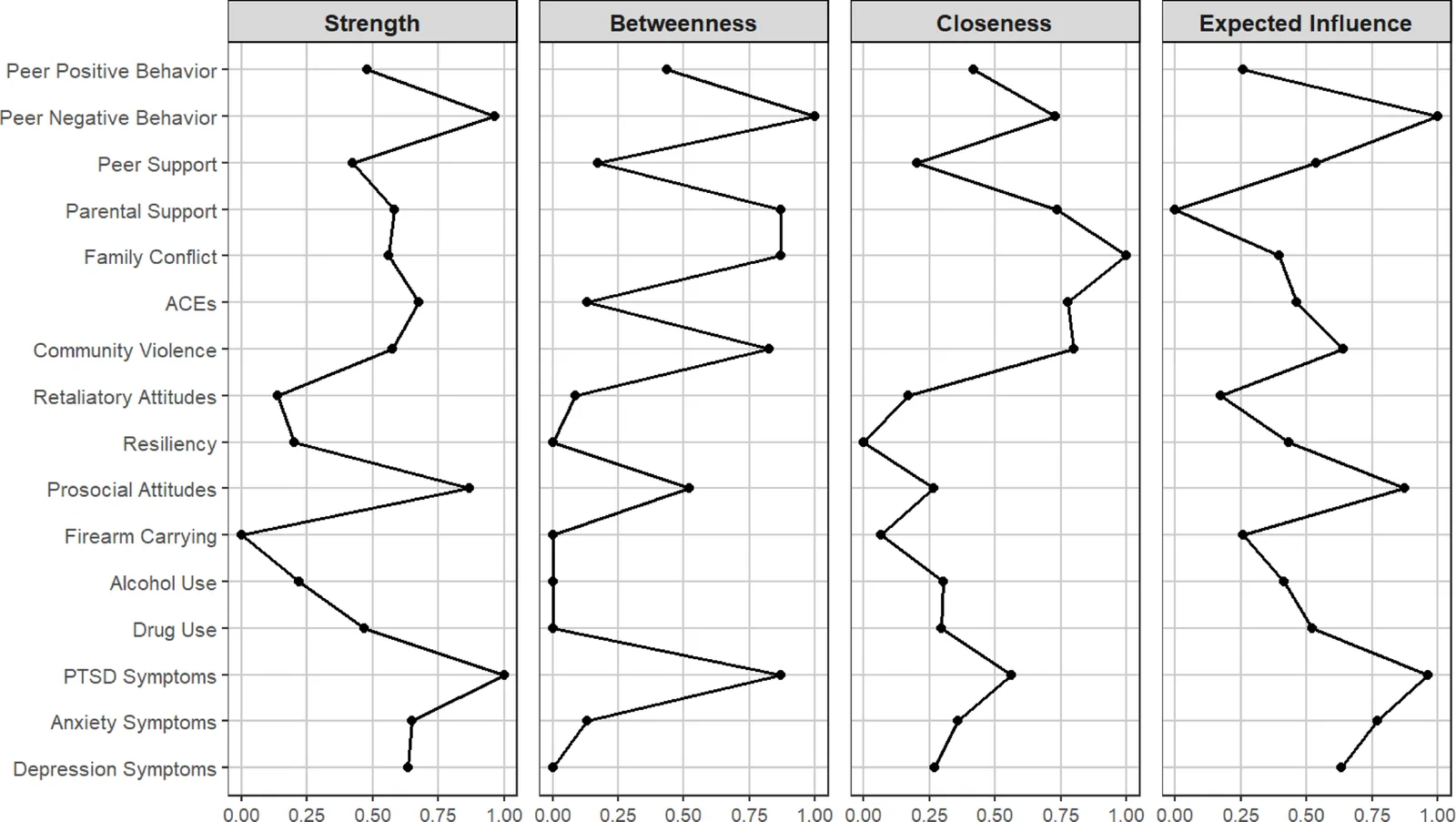
Four network centrality measures corresponding to each node in the correlation network of the factors potentially associated with firearm violence. All measures are calculated to be scaled between 0 and 1 (i.e., subtracting the minimum and dividing by the range). Values of one indicate the maximum on that measure, and zero indicates the minimum

Figure 2 presents multiple measures of network centrality. Community violence and family conflict showed relatively high betweenness and closeness, suggesting that they frequently lie along shortest paths and may function as bridging nodes connecting different domains of risk. Negative peer behaviors also exhibited high centrality across multiple measures, particularly strength, betweenness, and expected influence. PTSD had the largest absolute sum of edge lengths (strength) and the second-higher when sign was accounted for (expected influence); this, combined with its presence as a node among the higher values for Betweenness, suggests it is a highly central network element. Overall, these findings indicate that centrality metrics capture distinct aspects of network structure, including overall connectivity and positioning within paths between variables.

### Stability analysis

In Appendix Figs. 1–4 we present plots showing the correlation between the individual node-level centrality measures, and those estimated in repeated bootstrap resamples, as a function of the original dataset that was resampled. Both strength centrality and expected influence retained correlations > 0.9 even when the bootstrap sample size was only 25% of the original sample size. Closeness and betweenness both showed somewhat less stability, but retained high correlations (> 0.8) when the bootstrap sample size was under half of the original sample size, still well above suggested thresholds (i.e., > 0.7 when < 50% of the sample is used) for concluding good estimation stability.

### Discriminatory power

**Fig. 3:**
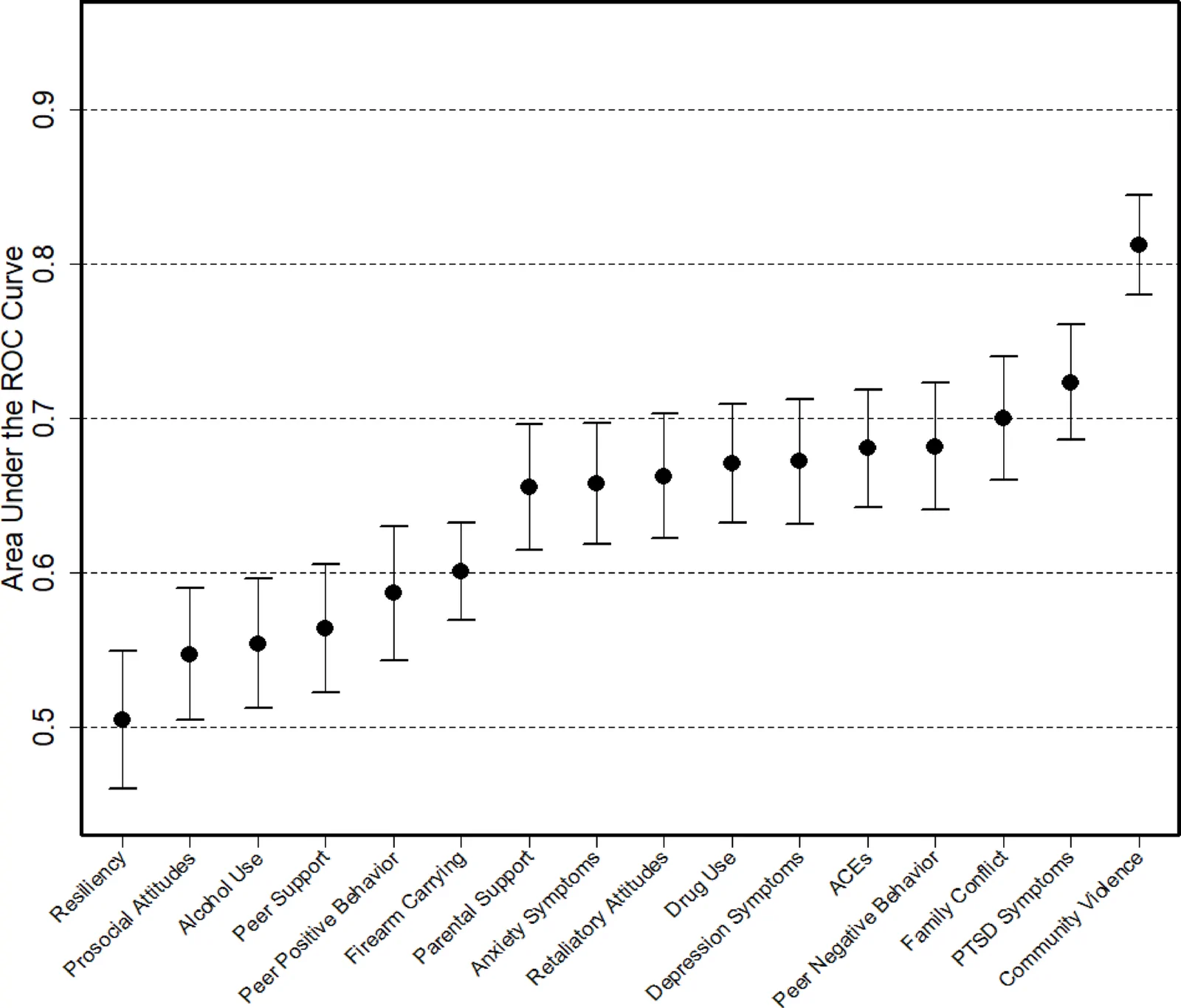
Area under the ROC curve displaying the power of each individual variable to discriminate between those with versus without firearm assault

Figure 3 shows the AUROC for each individual variable. Only resiliency and risky alcohol use produced non-significant (i.e., the CI covered 0.50) discriminatory power. A logistic regression model with all sixteen variables produces AUROC = 0.86 (95% CI 0.83, 0.89). Smaller AUROC values were seen more often among variables in the protective grouping (colored green), with the grouping collectively producing a logistic regression model with AUROC = 0.66 (95% CI 0.61, 0.70). Community violence exposure showed the best discriminatory power (AUROC = 0.81) by far; 93% of the AUROC of the full model. Over 96% of the discriminatory power—AUROC = 0.83 (95% CI 0.79, 0.86)—of the full model could be captured with the four consistently central network variables: PTSD, community violence exposure, negative peer behaviors, family conflict. Of note, firearm carrying frequency had significant, but modest, discriminatory power (AUROC = 0.60; 95% CI 0.57, 0.63).

## Discussion

This network analysis of SPARK data reveals that risk factors associated with firearm violence among young adults are not isolated, but form an interconnected network of mental health symptoms, substance use, peer relationships, adverse experiences, and protective influences. Centrality analyses identified PTSD, community violence, negative peer behaviors, and family conflict as structurally important elements within this network, and nearly the full discriminatory power of the complete model was preserved when focusing only on these most central factors. Notably, community violence exposure demonstrated the strongest individual discriminatory ability for firearm violence. This finding is consistent with prior SPARK analyses (Goldstick et al., 2024a, 2024b), which showed that community violence exposure had the largest adjusted association with firearm assault. Together, these results suggest that community violence exposure functions as a key marker of elevated risk, which may be due in part to its association with other factors.

The importance of community violence exposure highlights the role of broader structural and environmental conditions in shaping firearm violence risk. Structural racism, manifested through residential segregation, economic disinvestment, and discriminatory policies, concentrates community violence and limits access to resources in neighborhoods disproportionately populated by minority populations and marginalized identities (Bailey et al., 2017; Degli Esposti et al., 2022). Structural racism and discrimination not only increase the risk of violence exposure, but also lead to cumulative trauma, elevated stress responses, and heightened rates of mental health disorders like anxiety and depression (Bailey et al., 2017). Thus, addressing community violence exposure is critical to addressing firearm injury health disparities.

Interpersonal dynamics also play a significant role in shaping firearm violence risk. Negative peer behavior and family conflict were highly central and predictive for firearm violence risk. These social dynamics not only directly relate to firearm violence risk, but are related to other risk factors within the network, such as community violence (directly connected to negative peer behaviors in the network) and PTSD (directly connected to family conflict in the network). Youth exposed to risk factors like community violence, or suffer from PTSD symptoms, may be less equipped to navigate those exposures in the context of negative peers or familial conflict. Interventions that incorporate peer group dynamics, parental support, and family dynamics may be beneficial, particularly when integrated into broader community-level strategies.

We acknowledge study limitations. First, the cross-sectional nature of the baseline network analysis limits our ability to determine causality or directionality within the network, or in the associations between the risk factors and firearm assault experience. Relatedly, Self-report measures may be subject to recall bias and underreporting of stigmatized behaviors, such as substance misuse and violence exposure. Although validated measures were used, the parameters and their categorical definitions were constrained by the available data, which may limit their ability to fully capture underlying constructs and affect generalizability. Third, network models estimate conditional associations rather than causal effects and may be sensitive to model specification, variable selection, and regularization choices which can influence edge estimation and centrality metrics. However, we note in our supplemental analysis that the centrality measure estimation—which was the focus of our results—showed strong stability across bootstrap resamples, even with a sampling fraction < 50%. Additionally, the study excluded patients who were unable to consent or had certain clinical presentations, which may further limit generalizability. At the same time, the multi-site nature of our data set gives a robust view of the associations examined. Finally, victimization and aggression were combined into a single indicator in our analyses. Although this is concordant with prior literature, and other works shows that violent aggression and victimization epidemiology show large overlap, we note that most victimization was more common in this sample and therefore an analysis focusing solely on aggression may have shown some different results.

Among the interconnected risk and protective factors for firearm violence, community violence and PTSD are among potentially high-leverage intervention points due to their centrality and strong predictive power. Trauma-informed approaches targeting PTSD symptoms may be relevant given the centrality of PTSD within the network. However, these findings do not imply a causal pathway, and PTSD may reflect a consequence of firearm assault exposure rather than a precursor. Further longitudinal studies are needed to clarify causal relationships and potential intervention effects. In addition, addressing the effects of community violence, such as enhancing neighborhood safety and community cohesion, may help reduce the risk of future firearm violence. Interventions aimed at reshaping harmful social norms, such as weapon-carrying among peers, may improve the overall risk landscape as well. These findings reinforce that individual interventions alone cannot address all risk factors, which highlights the need for multifaceted, system-level interventions that simultaneously address individual trauma, social influences, and structural inequities to reduce firearm violence and the associated health disparities.

## Figures and Tables

**Fig. 4:**
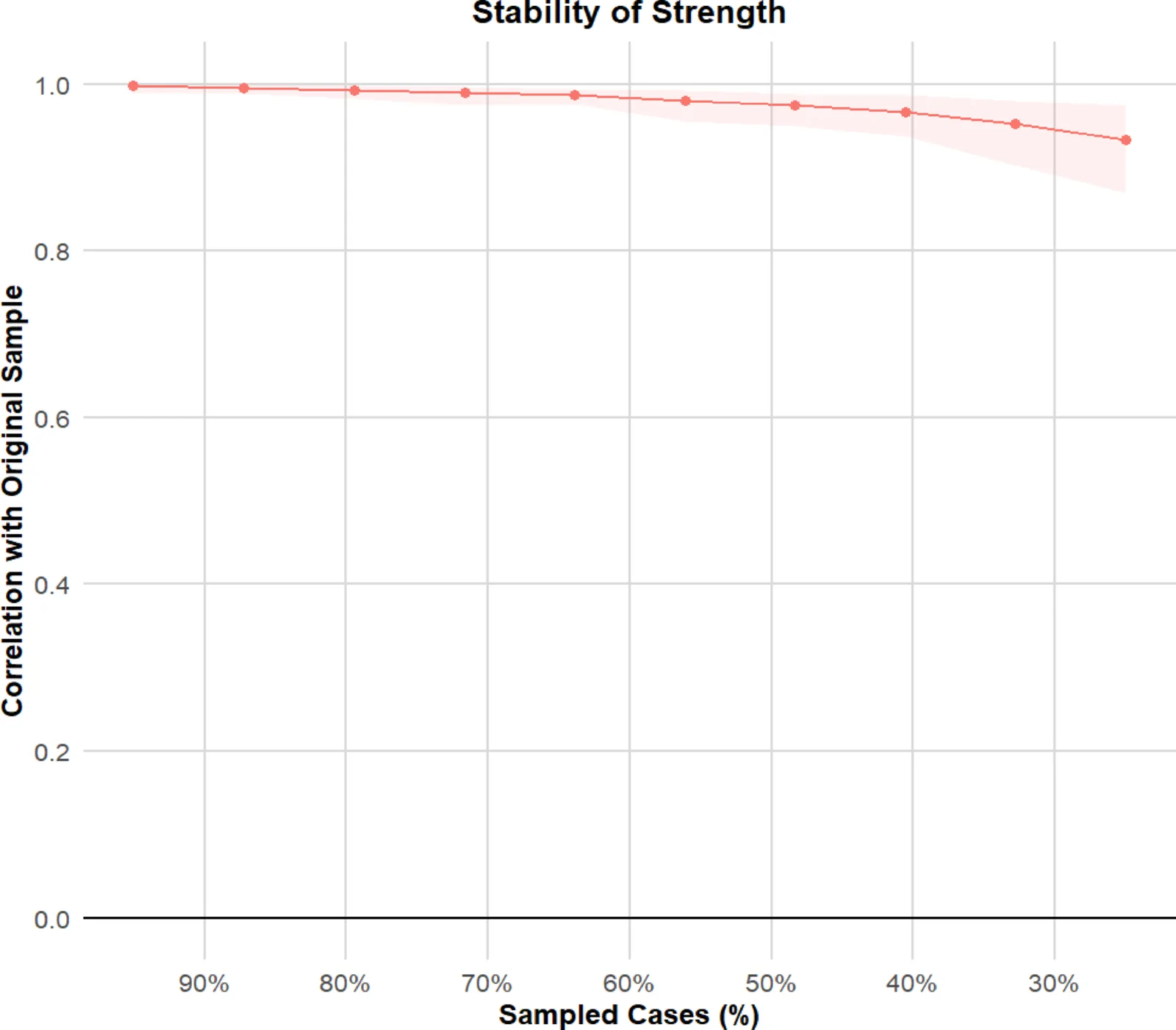
Stability of the strength centrality measure across n = 10,000 bootstrap resamples

**Fig. 5:**
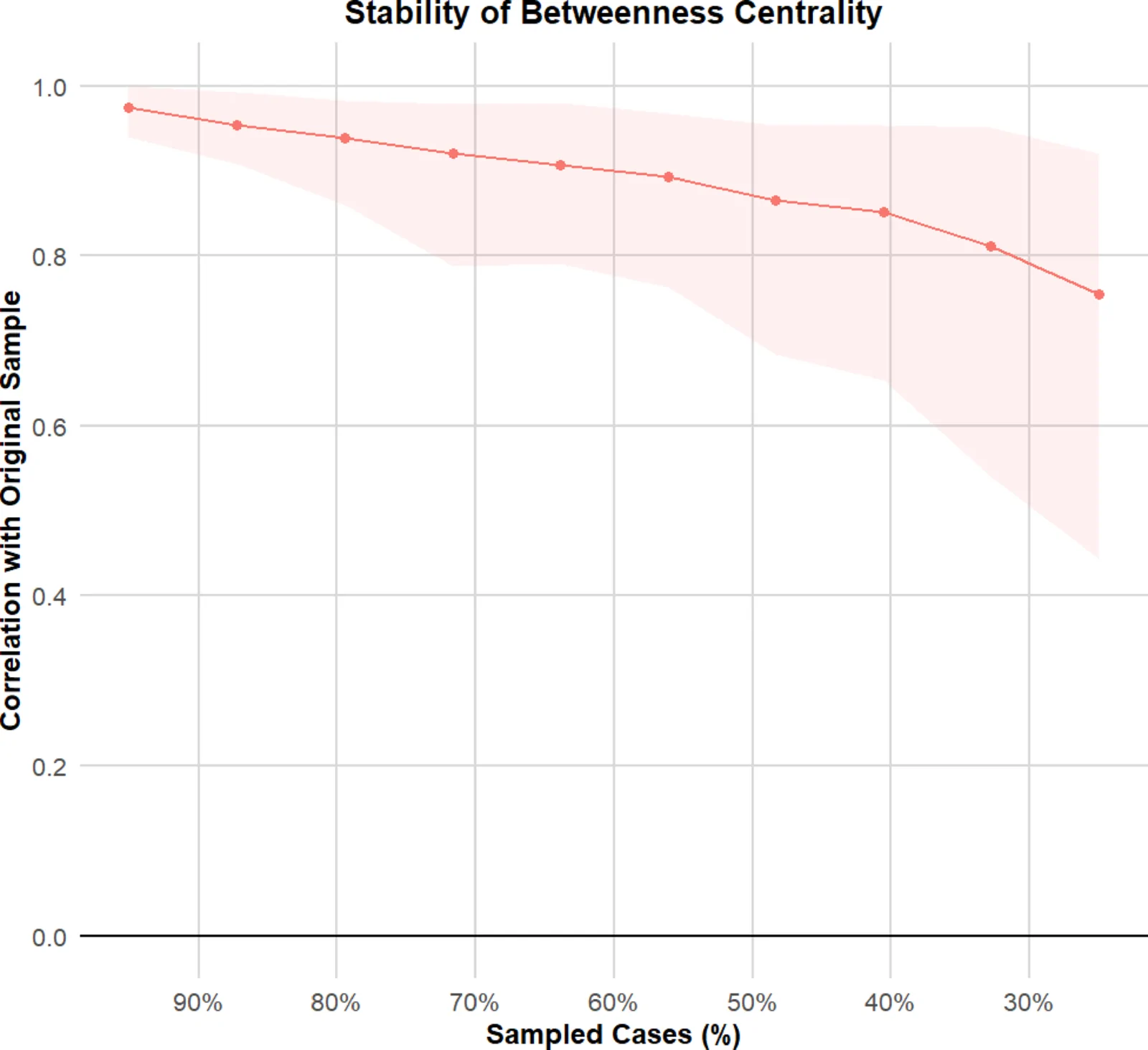
Stability of the betweenness centrality measure across n = 10,000 bootstrap resamples

**Fig. 6:**
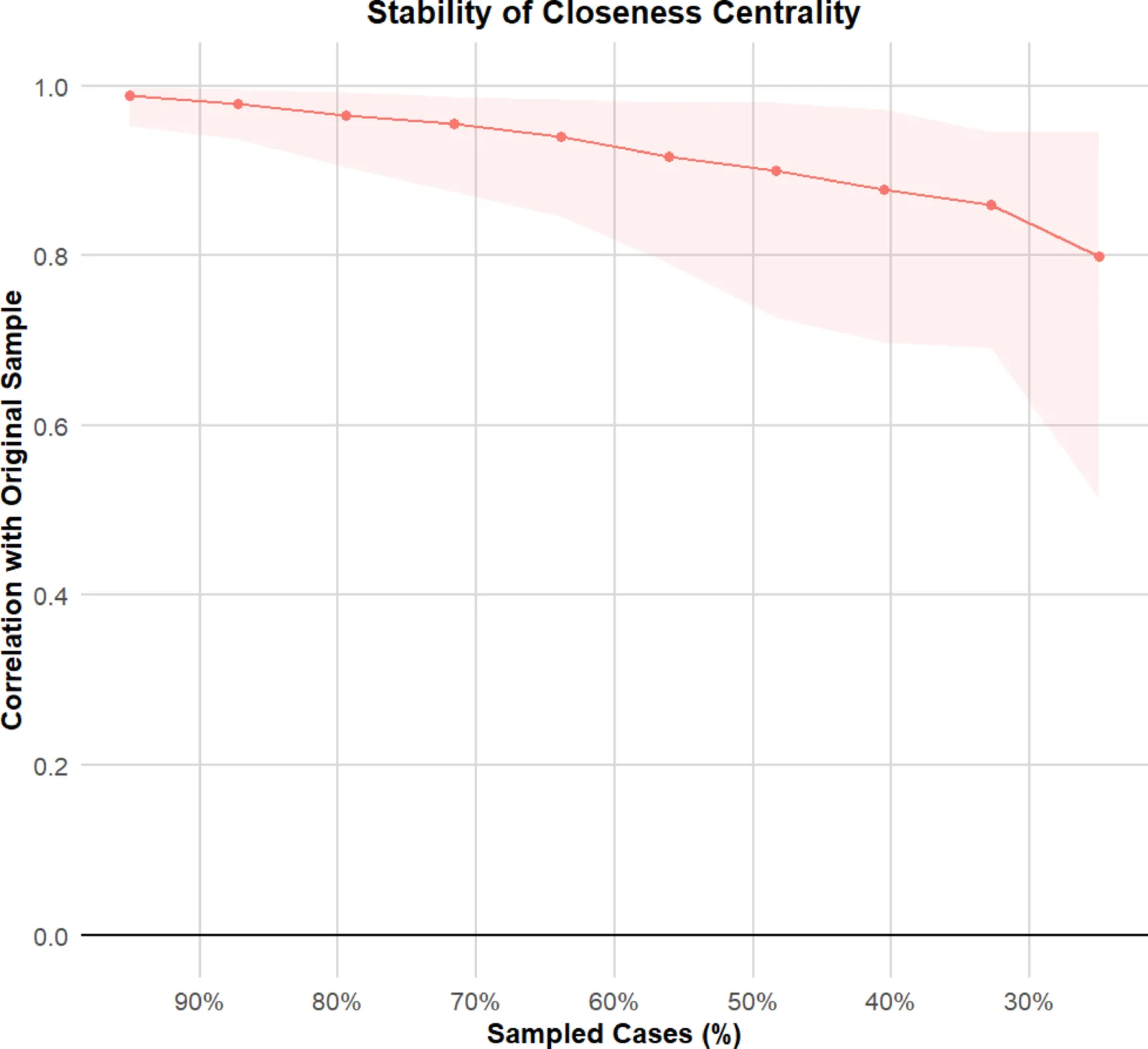
Stability of the closeness centrality measure across n = 10,000 bootstrap resamples

**Fig. 7:**
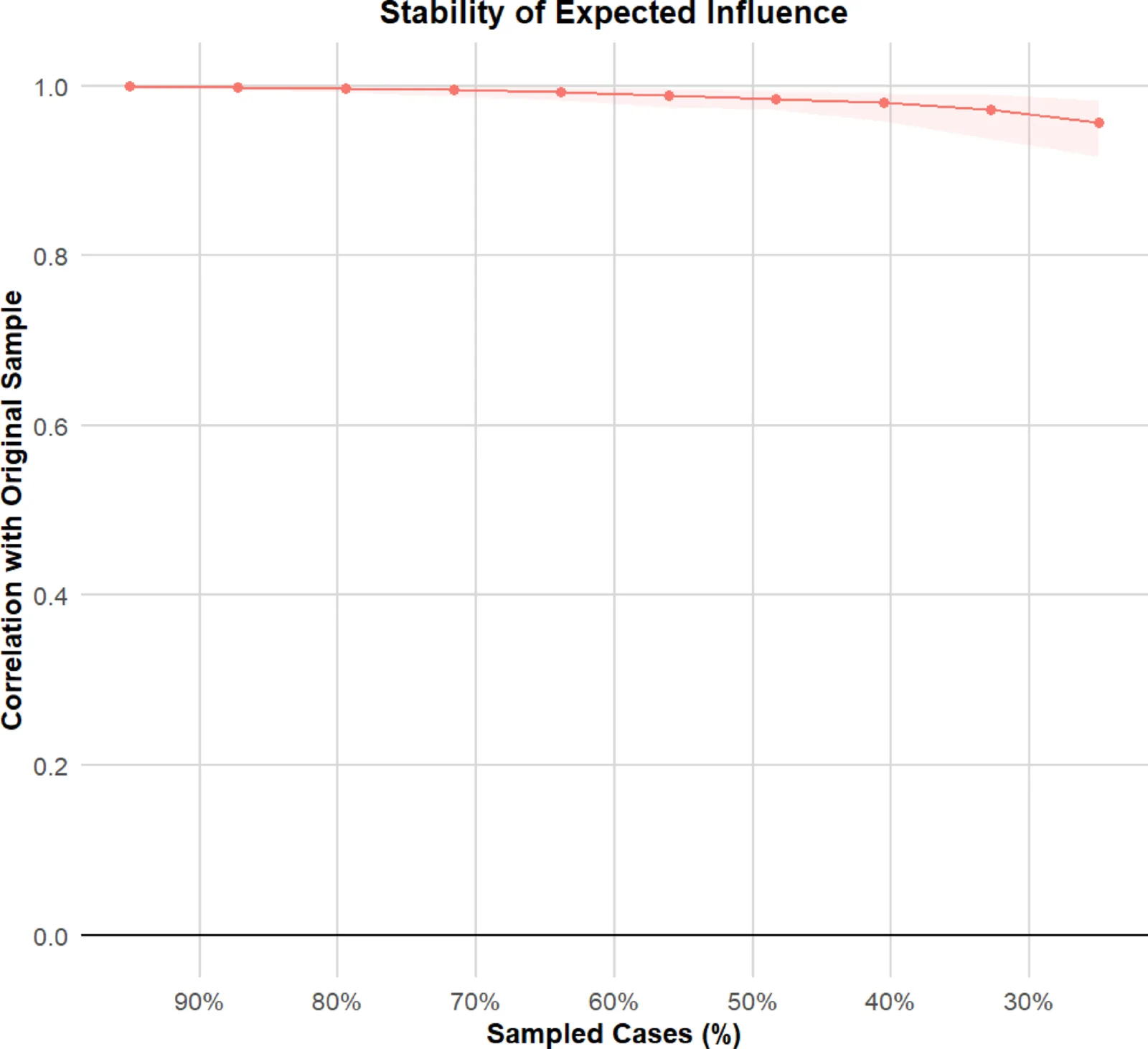
Stability of the expected influence centrality measure across n = 10,000 bootstrap resamples

## Data Availability

Available upon request.

## Appendix

See Figs. 4, 5, 6 and 7.
